# Establishing Healthy Eating ‘Habits’: A Pilot Randomised Controlled Trial of a Habit-Based Dietary Intervention following Oral Rehabilitation for Older Adults

**DOI:** 10.3390/nu15030731

**Published:** 2023-02-01

**Authors:** Sinead Watson, Leigh-Ann McCrum, Bernadette McGuinness, Christopher Cardwell, Mike Clarke, Jayne V. Woodside, Gerry McKenna, Laura McGowan

**Affiliations:** 1 Centre for Public Health, School of Medicine, Dentistry and Biomedical Sciences, Queen’s University Belfast, Belfast BT12 6BA, UK; 2 Institute for Global Food Security, School of Biological Sciences, Queen’s University Belfast, Belfast BT12 6BA, UK

**Keywords:** behaviour change, diet, intervention, randomised controlled trial, habit, healthy eating, older adults

## Abstract

An ageing population presents significant nutritional challenges, particularly for partially dentate adults. This two-armed pilot randomised controlled trial (RCT) compared habit formation (automaticity) for healthy eating behaviours between control and intervention groups after participation in a habit-based dietary intervention for older adults, following oral rehabilitation in the United Kingdom (UK). *n* = 54 participants were randomised to receive a habit-based dietary intervention (intervention group *n* = 27, IG) or standard dietary advice in a leaflet (control group *n* = 27, CG). The IG attended three sessions over six weeks, which focused on habit formation for three healthy eating behaviours (increasing fruit and vegetables, wholegrains, and healthy proteins). Participants were assessed for habit strength (using the Self-Report Behavioural Automaticity Index (SRBAI)) alongside health and nutrition outcomes at six weeks, four months and eight months. Forty-nine participants completed all follow-up visits. The IG compared to the CG had significant increases in automaticity at six weeks, four months (primary outcome) and eight months for eating ≥3 portions of fruit and vegetables; choosing wholegrain sources over white alternatives, and choosing healthy protein sources over red/processed meat. The mean change in the Mini Nutritional Assessment total score was greater in the IG compared with the CG at six weeks only (*p* = 0.03). A habit-based dietary intervention following oral rehabilitation increased automaticity for healthy dietary behaviours, which could translate into clinically meaningful benefits in this cohort of older adults.

## 1. Introduction

The ageing population in the United Kingdom (UK) presents significant nutritional challenges, where vulnerabilities to obesity, malnutrition and age related diseases are exacerbated by impaired dental and oral health status [1,2]. As natural teeth are lost, many older adults choose softer, more manageable foods often lacking in essential micronutrients and fibre [3,4]. Yet, replacing missing natural teeth alone does not positively influence their diet, demonstrating the need for intentional dietary intervention alongside tooth restoration in later life [5]. This ‘dual intervention’ approach of dietary intervention in combination with (or following) treatment to replace missing teeth (i.e., oral rehabilitation) is growing, though recent systematic review evidence highlights ambiguity regarding effective behavioural intervention components and outcomes, and a lack of theoretical grounding for the majority of existing interventions [6]. Dietary interventions with older people also appear to be short in duration and have unclear sustainability with regard to behaviour change maintenance [7,8]. 

Systematic review evidence suggests that the compelling issue of behaviour change maintenance within dietary interventions is not receiving the attention that it is due, yet sustained behaviours are vital if they are to produce meaningful changes in relevant health outcomes [9]. A plausible explanation to the shortcoming of behaviour sustainability is that interventions often focus on initiation factors that consist of reflective, conscious and deliberative processes. Yet, these first initiation factors differ from maintenance factors, and are not sufficient for the sustained engagement of a behaviour [10]. Habit formation has received interest across a variety of health behaviours due to a number of characteristics which make it appealing for understanding and modifying health behaviour [11,12,12,13]. Habit formation has previously been characterised as a simple, yet theoretically strong approach to modifying dietary behaviours within behavioural interventions [14,15,16,17] via repetition of a behaviour in a specific context [11]. Specifically, behaviour is prompted or triggered automatically via situational cues as a result of learned cue–behaviour associations [18]. 

Indeed, humans have been shown to automatically make over 200 food decisions each day, making it particularly interesting to investigate whether habit formation can facilitate healthful, sustained dietary behaviours [19], especially with older adults whom are likely to have established many habitual food-related behaviours over the life-course. A further benefit of creating habitual health-promoting behaviours relates to the theoretical suggestion that they can persist over time, supporting behaviour change maintenance. This stems from cue-action associations being strengthened each time the cue is encountered and the habitual impulse is enacted [20], leading to behaviours acquiring ‘automaticity’, i.e., feeling ‘automatic’ or ‘second nature’. 

There have been a number of studies to date which have primarily employed a habit formation approach to achieving positive dietary changes in a range of different population groups [14,15,16,17,21], yet, it has not been applied to dietary behaviour change interventions with older adults. The impact of small but sustained positive dietary behaviour changes which are facilitated by habit formation could be translated into a greater life expectancy and overall improved quality of life for older adults [22]. Furthermore, the impulse to act which is triggered from a learned cue behaviour context over time (without the need for additional monitoring or planning) could help to reduce the cognitive demand for enacting healthful dietary behaviours, particularly in ageing population groups where the risk of cognitive impairment is increased [23]. Habit theory has been used to develop behaviour change interventions with older adults to reduce sedentary behaviour and was considered a highly acceptable and feasible approach with this ageing population group [24].

The aim of this pilot randomised controlled trial (RCT) was to compare habit strength for three novel dietary behaviours (i.e., increasing consumption of fruit and vegetables (FV); increasing wholegrains; and, increasing healthy proteins) following a tailored, habit-based dietary intervention (versus control group) with older adults who had recently undergone oral rehabilitation. Changes to nutrient intakes (such as protein and fibre) were also examined as secondary outcomes (in Supplementary Files), as the focus for this trial was on establishing habitual healthy behaviours.

## 2. Materials and Methods

This pilot RCT is reported in line with the CONSORT and TIDieR checklist [25,26]. The trial was registered with the International Standard Randomised Controlled Trials Number registry (ISRCTN66118345) prior to participant recruitment.

The habit-based dietary intervention was developed in line with the Medical Research Council’s Guidelines for Developing Complex Interventions [27] which consisted of three distinct phases:

*Phase 1* involved analysing dietary intake of the target population using data from the UK National Diet and Nutrition Survey (NDNS), years 1 to 6 combined [4]. The NDNS generates publicly available, cross-sectional survey data, undertaken with a representative sample of people living across the UK. An analysis undertaken by our study team and published previously [4] informed the development of this dietary intervention focused on changing dietary behaviours that were most relevant to older adults’ nutritional needs. It indicated that the majority of older adults in the UK were *not* meeting: the 5-a-day recommendation for fruit and vegetables; the 18 g/day of dietary fibre (non-starch polysaccharides); and, the one portion (140 g) of oily fish per week recommendation. Focus group discussions with older adults (*n* = 21) also took place to gain input and feedback into the developed dietary intervention [28]. These discussions expanded upon the target dietary areas identified via the NDNS analysis, with amendments made as appropriate, for example, the oily fish recommendation was expanded to include a variety of healthy proteins (e.g., chicken, turkey, and eggs), as participants indicated this would be more attractive and achievable (reported previously [28]). 

*Phase 2* consisted of a small-scale, non-randomised (single-arm) study (*n* = 9) to test the feasibility of recruitment procedures and acceptability of the dietary intervention developed during Phase 1. Findings were very positive; participants found the intervention acceptable and useable with full details published previously [28]. Minor adjustments were made to recruitment procedures alongside refinements to the dietary intervention based on Phase 2 findings. 

*Phase 3* consisted of a single-site pilot RCT, the methodology and findings of which are reported below. This small-scale pilot RCT aimed to gather data to inform a definitive, multi-site RCT.

### 2.1. Recruitment

**Figure 1:**
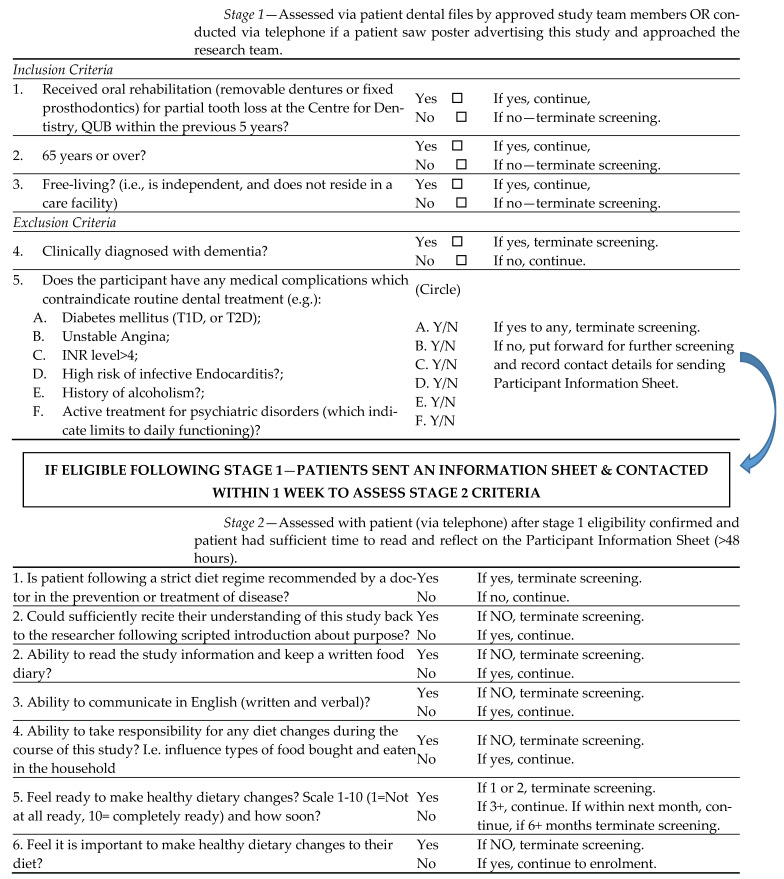
Screening Process DENHAB Intervention.

Dental patients who had completed oral rehabilitation for replacement of missing natural teeth and were under review in the Centre for Dentistry, Queen’s University Belfast were informed about this study (via letter, poster or in person at clinic appointments) and invited to participate. Potential participants were screened for eligibility in two stages detailed below and shown in Figure 1.

Patient dental files were screened for stage 1 eligibility under the following criteria: partially dentate with missing teeth replaced with removable partial dentures or fixed prosthodontics to provide a functional dentition within the last 5 years at the Centre for Dentistry; 65+ years; independent; no clinically diagnosed dementia; no diabetes mellitus; no history of alcoholism; no active treatment for psychiatric disorders; no medical complication which contraindicated routine dental treatment (see Figure 1).

If eligibility was confirmed after stage 1, the research team provided patients with a Participant Information Sheet. If patients expressed interest in this study directly (i.e., following seeing the poster in the clinic), the researcher confirmed stage 1 eligibility criteria with the patient, sent out an information sheet, and then re-contacted them >48 h after receiving the information sheet to assess stage 2 eligibility criteria. 

Stage 2 eligibility criteria were assessed via the telephone using the following key criteria (also Figure 1): not following a strict dietary regime recommended by a physician in the prevention or treatment of disease; could sufficiently recite their understanding of this study back to the researcher; ability to read the study information and keep a written food diary; ability to communicate in English; ability to take responsibility for any diet changes during the course of this study; ready to make healthy dietary changes; and found it important to make healthy dietary changes to their diet. If deemed eligible after stage 2 screening, those who were interested were given the opportunity to ask any questions/discuss the research in more detail and arrange the first study appointment. Or if patients preferred, the researcher contacted them again to confirm participation after a short period of time (if given permission to follow up). Written consent was obtained at the beginning of the first study visit. 

### 2.2. Study Design

A single-site (two arm) RCT was implemented between October 2017 and July 2019. Eligible participants were randomly assigned to intervention or control group directly following the collection of baseline research measurements. Randomisation to the tailored habit-based dietary intervention (intervention group) or minimal/educational intervention (control group) was coordinated by an independent statistician. The allocation sequence to intervention or control group was developed using a block randomisation approach stratified by gender (blocks size = 4) with computer-generated random numbers. Due to the nature of this study and the limited number of research staff, it was not possible to blind the participants or researchers to group allocation. The researchers were unaware of the randomisation schedule until after the baseline assessments when the sealed envelope containing the allocation outcome was opened. All participants were followed up at six weeks, four months and eight months after randomisation for assessment of primary and secondary outcomes. Study visits including assessments and intervention delivery were conducted either at the Centre of Dentistry, Queen’s University Belfast or in the homes of participants, if preferred. 

The control group was provided with publicly available written information containing current dietary advice at baseline (i.e., UK EatWell Guide leaflet) [29]. The researcher showed the participant the leaflet, briefly summarised the sections of the leaflet and suggested they read it in their own time. Weight, body mass index (BMI) and blood pressure information was given to participants at study assessment visits (if participant wished to know), although no detailed discussion took place based on these measurements, nor was dietary advice offered in the control group. Control group participants only had contact with the researcher at baseline and follow-up visits. 

The intervention group received the tailored, habit-based dietary intervention, as well as a copy of the UK EatWell Guide (discussed in the same manner as the control group) [29] (TIDieR—‘What’). The structure of the intervention was modelled on a previous RCT on creating healthy feeding (dietary) habits for parents of young children [14] and on feedback from the target population group during early intervention development [28]. The intervention was delivered face to face, using an intervention booklet (participant written materials), over three time points between baseline and 6 weeks (after trial baseline assessments were conducted) at fortnightly intervals. The researchers (qualified Nutritionist and Chartered Psychologist) were trained to deliver the intervention and collect research assessments, and followed a standardised protocol (TIDieR—‘Who and How’). Intervention participants received the same researcher for both the intervention delivery and research assessments. The intervention delivery component lasted approximately 30 min, which involved the researcher discussing the concept of habit formation with the patient, alongside introducing a different area of the diet to focus on at each of the three time points (fruit and vegetables, wholegrains or healthy proteins). A number of behaviour change techniques (BCTs) were included to support these healthy planned dietary behaviour changes during the habit acquisition phase. The specific BCTs employed have been coded from the BCT 93-item v1 Taxonomy [30] and are listed alongside a descriptor of how they were embedded within this study for both the intervention and control groups (Table 1) and the intervention group only (Table 2). 

A booklet was provided to participants and discussed at the first intervention appointment. The booklet included an introduction on how to create *‘healthy habits’* (language chosen for ease of participant understanding); tips on how to form *‘healthy habits’*; and detachable goal-setting and tracking sheets to self-monitor progress. A section on tips for healthy eating after oral rehabilitation was also introduced and discussed. There were separate sections for each of the three targeted dietary domains, which were introduced one at a time: at baseline visit—fruit and vegetables, at second visit (2 weeks after baseline)—wholegrains, and at third visit (4 weeks after baseline)—healthy proteins. An overview of the relevant targeted dietary domain was given (respective to each intervention session) which included information on recommended amounts (e.g., of fruit/vegetables to aim for); health benefits; good dietary sources; eating on a budget; portion guide; and a list of *‘healthy habits’* examples for ideas. Together with the researcher, participants decided on a tailored, novel, planned healthy behaviour to pursue in each of these three dietary areas (e.g., to have a banana with breakfast every day for fruit and vegetable domain). Participants then used the goal-setting sheets to write down when they were going to start making the planned dietary changes and identified any barriers, along with ways to overcome them. Exactly the same format was followed at each subsequent intervention delivery session, except that following the baseline visit, the researcher would use notes of the previous intervention session in the booklet to review progress towards the participants’ planned healthy behaviour (termed ‘*healthy habit*’) at the start of each visit. They would encourage the participant to continue carrying out their ongoing planned healthy behaviour(s) before introducing the next healthy eating domain (total of three) (TIDieR—‘When, How Much and Tailoring’).

### 2.3. Sample Size

The sample size of 25 patients per group (*n* = 50) was selected which would allow us 80% power to detect, as statistically significant at the 5% level, a difference in mean SRBAI between the intervention and control group with of 0.8 of a standard deviation (i.e., an effect size for SRBAI of 0.8). Data gathered from this single-site RCT will inform a definitive, multi-site RCT. 

### 2.4. Outcome Measures

Study assessments were conducted either at the Centre of Dentistry, Queen’s University Belfast or in homes of participants if preferred at baseline, six weeks, four months and eight months.

The primary outcome was change in self-reported behavioural automaticity index score (SRBAI) from baseline to four months between the intervention group and control group. This was assessed using the 4-item Self-Report Behavioural Automaticity Index (SRBAI) [31]. The four month time point was chosen in order to maximise the chances for the novel, planned healthy behaviours to acquire automaticity. The time taken to form health-related habits varies widely within the literature, i.e., from 18 to 254 days (average 66 days) [11]. Having a four month primary outcome assessment allowed for the six week intervention to be completed, plus allowing time for repetition of planned healthy behaviours in order to encourage formation of habitual behaviours. 

To allow for between-group differences to be examined, the intervention group and the control group completed a set of generically worded SRBAI items covering each of the three targeted healthy eating behaviours (i.e., fruit and vegetables, wholegrains, healthy proteins), based on previous research [4,14]. For each healthy eating behaviour, the 4 items of the SRBAI followed the same stem (e.g., “Choosing wholegrains, over white alternatives is something: I do automatically, I do without having to consciously remember, I do without thinking, I start doing before I realise I’m doing it). For fruit and vegetables the stem was “Eating 3 or more portions of fruit or vegetables each day is something…”. and for healthy proteins it was “Choosing healthier sources of protein instead of red or processed meats is something…”. Responses were measured using a 7-point Likert scale from 1 “strongly disagree” to 7 “strongly agree”. The scale also allowed a 0-point for ‘not relevant—I don’t do this regularly”. The 4 items of the questionnaire were averaged to give an automaticity score, taken as a measure of habit strength, for each healthy dietary behaviour domain. These generic SRBAI items were used to assess between-group differences in automaticity, with four months as the primary endpoint. 

For participants in the intervention group, the SRBAI was also used to quantify automaticity for their novel, planned healthy behaviours; however, the stem was tailored to fit the participant’s specific planned ‘*healthy habit*’. For example, if a participant set a novel healthy dietary behaviour goal such as ‘I will eat two vegetables with my main meal each evening’, this was inserted into the stem of the SRBAI to assess specific habit strength for that behaviour, such that it asked ‘Eating two vegetables with my main meal each evening is something I do…automatically, etc.’. The 4 items of the questionnaire were averaged to give an automaticity score for each healthy dietary behaviour domain (for the intervention group).

Secondary outcomes reported include the change from baseline to six weeks, four months and eight months between the intervention group and control group in the following measures:

### 2.5. The Mini Nutritional Assessment (MNA) 

Assessment of nutritional status was measured using the 18-item Mini Nutritional Assessment (MNA) questionnaire, which is recommended for people aged 65 years and older [32]. Each of the 18 items were weighted to calculate a total assessment score which was translated into three malnutrition indicator score categories: normal nutrition status (MNA score 24–30), at risk of malnutrition (MNA score 17–23.5), or malnourished (MNA score < 17).

### 2.6. Four-Day Food Diary

Dietary intake was assessed using a 4-day food diary, which involved participants recording everything they ate and drank over four consecutive days (including 1 day at the weekend). Participants were asked to estimate food and beverage portion sizes using household measures or through food and beverage labels. The researcher reviewed the diary for omissions and clarification at each visit (portion size, etc.). A nutrition analysis software programme called Nutritics (Research Edition, v5.09*, Dublin, Nutritics, 2019, https://www.nutritics.com/en/ accessed on 10 January 2019) provided estimates for daily energy and nutrient intakes from food and beverage sources (excluding supplements).

### 2.7. Other Health-Related Outcomes

Other health related outcomes assessed at baseline, six weeks, four months and eight months included, blood pressure (mm Hg) measured using an automated Omron sphygmomanometer and body mass index (BMI) calculated as weight (kg) divided by height squared (m^2^).

### 2.8. Other Measurements

At baseline, a questionnaire collected sociodemographic information including age, sex, ethnicity, occupation status, education and multiple deprivation score (MDS) [33], and lifestyle information including smoking status, alcohol consumption, physical activity, medication use, nutritional supplementation and oral health status.

In the intervention group, adherence to each planned dietary behaviour was assessed by the number of days out of 14 (two weeks) during the intervention period that participants reportedly carried out/enacted the planned dietary behaviour (termed ‘*healthy habit*’ for participants). Tracking sheets were provided to log progress each day, along with a pen and fridge magnet to ensure the self-monitoring sheet could be placed in a prominent position (e.g., stuck to the fridge). 

### 2.9. Statistical Analysis

SPSS Statistics for Windows version 25.0 (SPSS Inc., Chicago, IL, USA) was used for all analyses. Results are presented as the mean and standard deviations for continuous data and frequency and percentages for categorical data. The influence of the intervention on the primary (change in SRBAI for the three generic dietary behaviours from baseline to four months) and secondary endpoints was assessed by comparing the mean change in measurements from baseline to six weeks, four months and eight months between the intervention group and control group using independent-samples *t*-tests. Mean changes in automaticity scores for the three generic dietary behaviours (within the intervention and control groups) and the three individually tailored planned dietary behaviours (intervention group only) from baseline to six weeks, four months and eight months were examined using paired-samples *t*-tests. Spearman’s correlations were performed to investigate the associations between automaticity scores and the number of days participants carried out their healthy habits. A sensitivity analysis was performed on the MNA (rationale for this is provided in MNA results section).

## 3. Results

**Figure 2:**
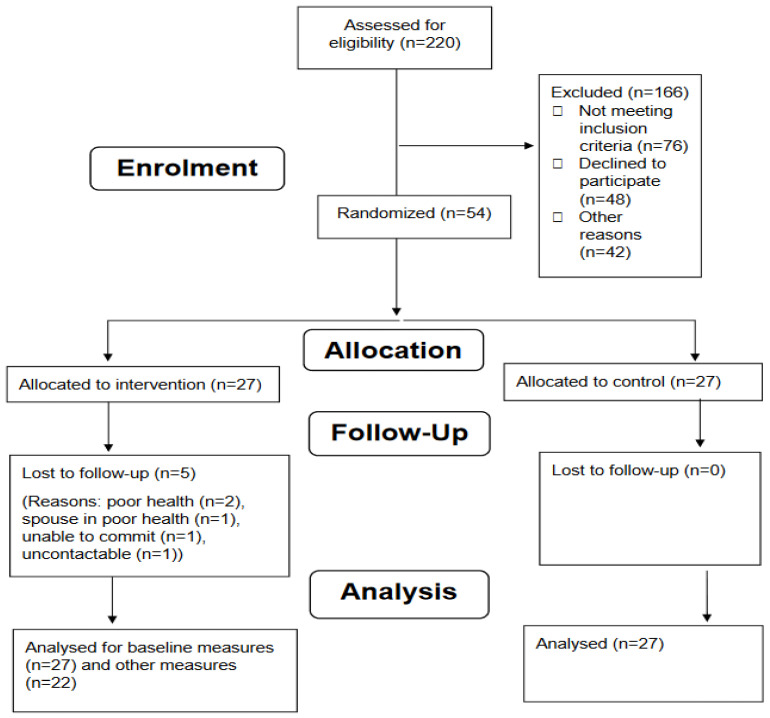
CONSORT Diagram Summarizing Flow of Participants’ SRBAI (automaticity) scores.

The recruitment pathway of this study is summarised in Figure 2 (CONSORT diagram). A total of 220 dental patients were screened for eligibility; 76 patients did not meet the inclusion criteria, and 48 patients declined to participate for the following reasons: no interest in changing diet (*n* = 22); poor health (*n* = 10); eat healthily already (*n* = 3); and too busy/too many commitments (*n* = 13). Other reasons for exclusion (*n* = 42) included uncontactable (*n* = 36); death (*n* = 1); reason unrecorded (*n* = 2); and dropouts prior to randomisation (*n* = 3). 

A total of 54 participants were randomised to the intervention (*n* = 27) or control (*n* = 27) group. Table 3 shows the baseline characteristics of the randomised sample. Five participants in the intervention group withdrew post-randomisation (dropped out during the intervention delivery period between weeks 2–6). A total of 49 participants completed all follow-up visits (22 intervention group and 27 control group), giving a 14% attrition rate. Those who completed the follow up visits had a higher baseline MDS (deprivation) score than those who withdrew from this study post randomisation (*p* < 0.05). No other differences in baseline characteristics were observed. Overall, 12 participants had their study visits conducted in their own homes with all others participating in study visits at the Centre for Dentistry. The majority of participants were of White ethnicity (94%). 

Table 4 shows the change in generic automaticity (SRBAI) scores for three generic dietary behaviours by randomisation group. No significant differences between randomisation groups were observed for the three dietary behaviours at baseline. There were significant increases in automaticity scores for the three dietary behaviours from baseline to each time point within the intervention group. Slight increases in automaticity scores for the three dietary behaviours observed in the control group from baseline to each time point were not statistically significant.

Between-group analyses using the generic SRBAI measure showed that the intervention group compared to the control group had significant increases in automaticity from baseline to the four month primary endpoint for eating ≥3 portions of fruit and veg (mean change [95% CIs]: −1.9 [−3.3, −0.6] *p* = 0.008); choosing wholegrain sources over white alternatives (mean change [95% CIs]: −2.6 [−3.9, −1.2] *p* < 0.001), and choosing healthy protein sources over red/processed meat (mean change [95% CIs]: −1.3 [−2.4, −0.2] *p* = 0.027). The intervention group compared to the control group additionally had significant increases in automaticity for the three dietary behaviours from baseline to six weeks and from baseline to eight months.

As shown in Table 5, increases in specific automaticity (SRBAI) scores for all three of the individually tailored planned dietary behaviours were observed within the intervention group from baseline to six weeks, baseline to four months and baseline to eight months.

Approximately, 64% of participants in the intervention group engaged with tracking their fruit and vegetable habit, 73% tracked their wholegrain habit and 59% tracked their healthy protein habit. Spearman’s correlation analyses demonstrated strong positive correlations between specific automaticity scores and the number of days participants carried out their healthy habits at 4 months (fruit and vegetables r = 0.74, *p* < 0.001; wholegrains r = 0.91, *p* < 0.001; healthy proteins r = 0.82, *p* < 0.001), and moderate to strong correlations at 8 months (fruit and vegetables r = 0.62 *p* = 0.002; wholegrains r = 0.59, *p* = 0.004; healthy proteins r = 0.9, *p* < 0.001). No significant correlations were identified at 6 weeks.

### Dietary Data

From baseline to 6 weeks, the intervention group significantly increased their daily mean intakes of protein (mean change [95% CIs]: 11.65 g/d [0.64, 22.67]; *p* = 0.04), fibre (4.06 g/d [0.40, 7.71]; (*p* = 0.03), folate (90.95 µg/d [6.52, 175.38]; *p* = 0.04), vitamin C (54.81 mg/d [6.07, 103.54]; *p* = 0.03), calcium (228.60 mg/d [36.92, 420.28]; *p* = 0.02), magnesium (59.07 mg/d [14.13, 104.01]; *p* = 0.01) and potassium (517.11 mg/d [117.81, 916.40]; *p* = 0.01) (all within-group pre–post changes). The control group’s mean daily protein and free sugar intake significantly decreased from baseline to 8 months (−6.10 g/d [−11.47, −0.72]; *p* = 0.03 and −9.86 g/d [−16.36, −3.35]; *p* = 0.01, respectively). No other significant differences in nutrient intakes across the time points within each group were observed.

The intervention group significantly increased their daily mean intake of potassium relative to the control group from baseline to 6 weeks (mean difference [95% CIs]: −452.40 [−15.61, −889.19]; *p* = 0.04). No other significant between-group differences in nutrient intakes across the time points were observed (all between-group nutrient data provided in Supplementary Tables S1 and S2). 

According to the MNA at baseline the majority of participants had a normal nutritional status apart from 3 (14%) participants in the intervention group and 4 (15%) participants in the control group who were at risk of malnutrition. At 6 weeks, nutritional improvements were observed, as none in the intervention group and only 2 (8%) in the control group were at risk of malnutrition. At 4 months, 1 (5%) participant in the intervention group was malnourished and 1 (4%) participant in the control group was at risk of malnutrition. At 8 months, the same participant (5%) in the intervention group remained malnourished, while 2 (7%) participants in the control group were classified as at risk of malnutrition.

Table 6 shows the mean change in MNA scores at each time point for the control and intervention groups. A positive change score is considered an improvement in nutritional status. The change from baseline in total score was greater in the intervention group compared with the control group at 6 weeks only (*p* = 0.03). A sensitivity analysis was conducted with the removal of an intervention group participant who was malnourished and facing significant personal challenges due to bereavement (although wished to remain in this study). Results demonstrated a significant difference at both 6 weeks (*p* = 0.040) and 4 months (*p* = 0.049) in favour of the intervention group. 

Changes in blood pressure (systolic and diastolic) and BMI from baseline to 6 weeks, 4 months and 8 months, were not significantly different between the intervention and control groups, and there were no significant differences in physical activity between the groups at any time point (data not shown). 

## 4. Discussion

Despite the need for novel, theory-based dietary interventions for older adults following oral rehabilitation, the utility of habit formation as a means of facilitating healthy dietary behaviours had not previously been applied to this population. This pilot RCT compared habit strength between intervention and control groups for three novel, planned dietary behaviours in older adults who had previously undergone oral rehabilitation. The mean change in automaticity scores for all three generic dietary behaviours (as measured by SRBAI) were consistently higher within the intervention group compared to the control group at 6 weeks, 4 months (primary end point) and 8 months. This suggests that participants’ self-reported increases in enacting healthy planned dietary behaviours with less conscious awareness (greater automaticity) in the areas of FV, fibre and healthy protein intake. 

For the intervention group, all three specific, planned healthy behaviours became substantially more habitual over the course of the intervention, as manifested by significantly higher automaticity scores from baseline to 6 weeks onwards. Following the principles of habit formation, i.e., repetition within the same context/in response to the same cue, the planned behaviours became increasingly automatic, in line with previous habit literature and showed the ability for older people to demonstrate healthy dietary behaviour change [11,12,14,34].

Across all three planned healthy behaviours, associations between automaticity and self-reported behavioural frequency were evident (i.e., self-reported adherence to carrying out the planned ‘healthy habit’). These measures can be considered predictors of one another; however, research suggests that although repetition (of the behaviour) is required for habits to develop, automaticity should be considered as a mental construct (lack of awareness, etc.), and more than just frequency of occurrence [35]. Although correlation cannot confer causality in the present study, it is suggestive that changes in behaviour were facilitated by changes (increases) in habit strength. This supports habit theory, where increased repetition of a behaviour in a stable context enables the development of automaticity [12,34,36]. Nonetheless, it was evident that habitual behaviours appeared to be enacted with the greatest automaticity by the 4 month study visit mark, as no significant habitual gains were observed between then and the final follow up visit (8 months). This appeared to be reflective of the development of habits as an asymptotic curve which has been previously reported, where greatest habit gains are observed early on, following which they plateau [11].

Research has shown that habit behaviour effects are mostly attributable to habitual instigation, and not execution, and forming habits for both instigating and executing a behaviour may help maximise the likelihood of maintenance for certain behaviours [37]. In the present research, the SRBAI, was used as the primary outcome, which is proposed to capture habitual instigation; it may be the case that greater behaviour change could be achieved by working on a series of preparatory and target behaviours for dietary changes where variety is not needed, e.g., having a glass of milk with dinner each evening [37]. However, for other healthy behaviours such as having two vegetables with lunch each day, dietary variety in the choice of vegetables would be recommended for health, therefore focusing mainly on habitual instigation is appropriate for this planned behaviour. The impact of increased automaticity for these positive dietary changes illustrate the potential impact this could have on healthy ageing.

A number of BCTs were included in the intervention to support healthy planned dietary behaviour changes during the habit acquisition phase. These BCTs including problem solving, use of follow-up prompts and goal setting (outcomes), have been used in other similar dietary intervention studies involving older adults, and appear to enhance the effectiveness of such interventions [38]. These BCTs may have facilitated behaviour change in synergy with and/or in addition to the process of habit formation, yet it was not possible in the present study to explore the relative contribution of each. Small gains in the control group in automaticity (not significant) may have been seen due to this group receiving ‘minimal intervention’, i.e., an educational leaflet, versus no intervention.

Previous qualitative research with older adults in the domain of physical activity showed that successful long-term users of wearable activity trackers typically described starting with a small behavioural goal and gradually increasing it, and action planning and coping planning strategies were evident [39]. These latter BCTs of action planning and coping planning were unique to the intervention group in the present study in relation to dietary changes, and may partly explain their increased success with behaviour changes. It should be noted that physical activity was not targeted in this pilot intervention as the focus was to deliver a behavioural intervention to establish habitual, healthy dietary behaviours following oral habilitation. This grew from clinical and research evidence highlighting an area of nutritional need in those who have undergone oral rehabilitation, and was not designed as a weight management intervention where diet and physical activity would typically be targeted simultaneously. However, the majority of adults recruited had overweight or obesity and the inclusion of physical activity and strength training is an important aspect to consider in a future intervention, particularly as many BCTs will overlap between diet and activity behaviours. This is particularly pertinent given the growing challenge of sarcopenic obesity (new class of obesity in older adults in which low skeletal muscle mass is coupled with high levels of adiposity) [40]. Furthermore, it is suggested that diverse mechanisms, beyond habit formation, should be considered in behaviour change interventions [41]. One mechanism which may be relevant to the present dietary intervention is that of ‘taste discovery’, i.e., whereby after repeatedly eating wholemeal bread, or eating novel fruit and vegetables in the habit acquisition phase, an individual learns that they like this food (which drives future consumption) [41]. Future process evaluation of interventions with this population group will help delineate these influences on behaviour change.

Some improvements in nutrient intakes in the intervention group were observed during this study. However, when compared with the control group, the intervention group only had a significantly higher mean change in potassium from baseline to 6 weeks, despite protein and fibre approaching significance at this time point indicating positive trends. Similarly, calcium showed a positive trend between groups at 8 months (non-significant), and increases in the intervention group may have been explained in part by many participants choosing to increase their milk intake as part of the healthy protein habit. The change in total MNA score from baseline to 6 weeks, however, was greater in the intervention group compared with the control group. It is important to consider that this is a pilot study and therefore the sample size may have been too small to detect a significant difference between groups for some of the outcomes such as dietary intake. Furthermore, despite these significant improvements in total MNA in the intervention group relative to the control group, it was questioned if the MNA was a valid tool for assessing malnutrition or truly captured nutritional change amongst the community dwelling older people of this study, despite it being the most recognised nutrition screening tool currently available [42]. For example, the ambiguity of this study’s effect using the tool was notable, particularly as many participants reported that they ate healthfully already (despite study screening measures, discussed further below), meaning optimal diet scores in many cases were already observed. Additionally, diet-related questions were not all necessarily directly influenced (i.e., targeted) by the healthy eating domains within this specific intervention. The present study opted for a 4-day food diary including weekdays and weekends (alongside the MNA) to assess diet (tested for acceptability in Phase 2 [28]) to allow investigation of the specific nutrients targeted in the intervention as secondary outcomes. This followed a similar approach taken in previous interventions, which used 3- or 4-day food diaries for dietary assessment. [43,44] There remains limited consistency regarding nutritional assessment and scoring and/or use of healthy eating indexes within the current literature for older adults who have undergone oral rehabilitation, particularly in the UK, which is outlined in a paper by Moynihan and colleagues (2009) as a methodological limitation in the field. [45] Although the generation of an index or healthy eating score was beyond the scope of the present research, it is certainly possible in the future given the data available from the 4-day food diaries. Consideration should also be given to assessing and benchmarking dietary status in older adults, across countries (most tend to focus on US dietary recommendations [46]), with a suitable instrument which balances participant burden and research needs (such as sensitivity, comprehensiveness, and comparison between countries). 

There are several strengths to this study, including the use of a theory-based intervention with a robust RCT method at a single site. This study is believed to be the first of its kind to follow the MRC guidelines for developing complex interventions within oral rehabilitation research and is one of a small number of nutrition studies that accounts for oral health in this population. There are a number of limitations to this research as well, including the use of a self-reported habit automaticity measure. It is debated whether individuals have sufficient ability to accurately self-report on habit strength when it requires reflection upon a non-reflective process [47,48]. However, as the most established measure of automaticity to date, it is currently the best suited measure for the purpose of this study and does allow for comparability between other research studies. As self-reporting automaticity may convey perception of change, instead of actual change, habit literature may benefit if future research was to focus on alternative measures of automaticity, which are accessible for participants, particularly in this age group. Furthermore, the sample size may not have been large enough to adequately detect clinically meaningful differences in dietary intake as it was based upon detecting a difference in automaticity (SRBAI). The effect size anticipated in this trial was large (0.8), and the observed SRBAI effect size was large. It could be argued that whilst this measure helps elucidate understanding around potential mechanism of behaviour change (i.e., habit strength), it is more difficult to assign a clinically meaningful change in SRBAI score and future research may wish to take this into consideration. 

Differential attrition was noted between the intervention and control groups, with *n* = 5 participants lost to follow up within the intervention group, and none lost within the control group. This may have been in part due to the two extra visits which intervention group participants received for intervention delivery, which for some in this age group proved a challenge. Other reasons were beyond the scope of this study and unavoidable, e.g., caring responsibilities and poor health. It was also not possible to conduct blind study assessments for either control or intervention groups, for example, in the control group the same researcher introduced the leaflet containing publicly available dietary advice and conducted study assessments. Similarly, in the intervention group, participants received the same researcher for intervention delivery and collection of research measures (they were also provided with the leaflet containing publicly available dietary advice in the same way as the control group); however, this may have introduced bias via social desirability in participant responding and in assessments. Furthermore, two researchers were involved in the delivery of both control and intervention sessions (as this was randomly assigned following baseline assessment), although every effort was made to ensure consistency by following a detailed protocol for all study visits (across control and intervention) and undertaking training in intervention delivery specifically. 

Certain groups within the older adult population may have been under-represented, such as ethnic minorities (a predominantly White population engaged), those with a low BMI, and those from deprived backgrounds (discussed further below). Unfortunately, this may limit the representativeness of the pilot RCT findings to the general population beyond Northern Ireland; despite closely reflecting the ethnicity profile of Northern Ireland within the UK (with 96.6% of the population classified as White in the 2021 census [49]), where this study was conducted. Other factors such as willingness to perform the research assessments, and willingness to make healthy dietary changes should also be considered regarding generalisability of the population within this intervention as recruitment methods meant only motivated individuals were enrolled into the behavioural intervention, and they had to self-report a willingness to make dietary changes (screening issues relating to dietary assessment are discussed further below). This means that those who did not consciously identify (or prioritise) a need to make dietary changes may have been under-represented, despite NDNS data suggesting that the majority of older adults could benefit from this type of intervention [4]. 

Considerations for future research arising from this pilot study relate to the family structure and living arrangements of the participants, which can directly influence responsibility for food/dietary choices. Participants in this study were asked about living status (i.e., they needed to be free-living, not residing in a care facility) and they were only included if they agreed that they could influence the types of foods bought and eaten within their household as the intervention involved working directly with them on a one-to-one basis. Previous research has shown the importance of social support as a behaviour change technique for increasing FV intake via dietary interventions [38], and therefore testing this approach with other family structures is of interest, i.e., working with dietary gatekeepers for older adults (whether family members or within care settings), or couples within a household, etc. This type of habit-based intervention may indeed be applicable across alternative living arrangements, as previous research showed positive changes to the dietary intake of young children when parents were targeted as the nutritional gatekeepers, in the habit-based intervention which informed the present study [14]. Furthermore, there are broad considerations regarding recruiting older adults to a dietary intervention such as this, as screening and measuring baseline eating habits/dietary quality can often be challenging [50]. For example, in the present study, participants were given information on the aim of this study (making healthy changes to diet) and asked about their readiness and motivation for making healthy dietary changes with a small number (*n* = 3) declining to participate as they felt they ate healthily already. Participants were only included if they felt somewhat ready to make changes and that it was at least somewhat important to them. This was felt necessary for the present behavioural intervention, and differing interventions may include different strategies for recruiting participants at different levels of readiness for behaviour change. Most participants had overweight or obesity in the present research and felt they could make improvements to their diet, although at baseline the majority of participants had a normal nutritional status as assessed via the MNA (limitations noted above). It was considered that the inclusion of a brief validated dietary screening measure would allow greater stratification of nutritional need and perhaps allow a more targeted approach to enrolling those most at risk. It is also worth noting that the present sample was skewed towards those characterised as least deprived (as measured by the MDS), and this may limit the generalisability of the intervention across the socioeconomic spectrum, particularly as those living in areas of greater deprivation are less likely to meet healthy eating guidelines, particularly for FV and fibre [51].

## 5. Conclusions

The results of this trial are supportive of the role that habit formation can play in achieving increased habit strength for novel, healthy planned dietary behaviours. This pilot study should now form the basis of a larger, multi-site trial that will continue to advance the science of habit-based behaviour change interventions as a means of facilitating healthful dietary change in older adults, and crucially, promote long-term behavioural maintenance. Furthermore, as previous research demonstrates, replacing missing teeth alone does not positively promote healthy changes in diet in older adults [5], future studies should, therefore, explore the potential of implementing such dietary interventions in the dental care setting, within a multidisciplinary approach to improving patient health behaviours and nutritional status.

## Figures and Tables

**Table 1:** BCTs for Intervention and Control Groups using the 93 Item V1 Taxonomy.

BCT	BCT Definition	BCT Descriptor in This Study
**Biofeedback**	Provide feedback about the body using an external monitoring device as part of a behaviour change strategy	Giving feedback of changes in anthropometry and other health outcome measures in comparison to previous study visits at each assessment (e.g., weight and BMI) ^†^ (N.B. This was an active part of the intervention for the intervention group; it occurred only via data collection procedures for the control group, and feedback was only given if requested by participants; nonetheless it may have influenced the behaviour of control group participants, hence listed here)
**Credible source**	Present verbal or visual communication from a credible source in favour of or against the behaviour	Health information given out by a trained researcher in the form of the EatWell Guide leaflet and also through giving anthropometrics data and other health outcome measures ^†‡^
**Information about health consequences**	Provide information (e.g., written, verbal, visual) about health consequences of performing the behaviour	Health information given via the EatWell Guide leaflet^‡^
**Monitoring outcome(s) of behaviour by others without feedback**	Observe or record outcomes of behaviour with the person’s knowledge as part of a behaviour change strategy	Recording of anthropometry and other health outcome measures at each assessment ^‡^ (N.B. This was an active part of the intervention for the intervention group; it occurred only via data collection procedures for the control group, but nonetheless may have influenced the behaviour of control group participants, hence listed here)

^†^ BCT primary mode of delivery—face to face. ^‡^ BCT primary mode of delivery—written.

**Table 2:** BCTs for Intervention Group only using the 93 Item V1 Taxonomy.

BCT	BCT Definition	BCT Descriptor
**Action planning**	Prompt detailed planning of performance of the behaviour (must include at least one of context, frequency, duration and intensity). Context may be environmental (physical or social) or internal	A planning sheet included in the intervention booklet where participants write down their healthy habits along with where and when they would perform the behaviour as well as preparation and start dates ^†,‡^
**Behaviour substitution**	Prompt substitution of the unwanted behaviour with a wanted or neutral behaviour	In some scenarios a good behaviour was substituted for a bad behaviour e.g replacing an unhealthy dessert with fruit ^†^
**Behavioural practice/rehearsal**	Prompt practice or rehearsal of the performance of the behaviour one or more times in a context or at a time when the performance may not be necessary, in order to increase habit and skill	Habit was practiced/rehearsed by repeating in the same context and potentially in other contexts ^†,‡^ (N.B. also coded alongside ‘habit formation’ as per suggestion of Michie et al., 2013) [30] In practice, this could have been achieved by the participant choosing to eat wholegrain bread for breakfast, yet their specific planned behaviour was to eat wholegrain bread for lunch each day; practicing eating wholegrain bread at other times of day may have facilitated the development of habitual wholegrain dietary choices/intake at lunch.)
**Feedback on behaviour**	Monitor and provide informative or evaluative feedback on performance of the behaviour	Giving feedback on performance of the behaviour of previous study visits at each assessment
**Goal setting (behaviour)**	Set or agree on a goal defined in terms of the behaviour to be achieved	A planning sheet was included in the intervention booklet where participants discussed and wrote down their goals ^†,‡^
**Goal setting (outcome)**	Set or agree on a goal defined in terms of a positive outcome of wanted behaviour	To increase fruit and vegetable, wholegrain and healthy protein intake ^†^
**Habit formation**	Prompt rehearsal and repetition of the behaviour in the same context repeatedly so that the context elicits the behaviour	Three planned, novel healthy dietary behaviours were rehearsed by repeating in the same context in order to establish habitual behaviour which becomes automatically cued upon encountering the specific associated cue ^†,‡^
**Habit reversal**	Prompt rehearsal and repetition of an alternative behaviour to replace an unwanted habitual behaviour	Some unwanted behaviours were specifically identified and substituted e.g the swapping of an unhealthy habit for a healthy habit e.g fizzy drink for milk ^†,‡^
**Instruction on how to perform a behaviour**	Advise or agree on how to perform the behaviour	Instructions given as sections such as portion guide and eating on a budget were discussed ^†,‡^
**Problem solving**	Analyse, or prompt the person to analyse, factors influencing the behaviour and generate or select strategies that include overcoming barriers and/or increasing facilitators	Participants were prompted to identify barriers that might get in the way of making successful changes, along with methods to help prevent them affecting habit formation. These were then written in the intervention booklet ^†,‡^
**Prompts/cues**	Introduce or define environmental or social stimulus with the purpose of prompting or cueing the behaviour. The prompt or cue would normally occur at the time or place of performance	Participants were asked to define a context in which to perform the behaviour which was then their cue. For example, if the participant chose a novel planned behaviour such as ‘having a glass of milk with lunch each day’ then ‘lunch’ become the context which was then their cue to have the glass of milk. Tracking sheets and fridge magnets were also provided which may have acted as a visual cue to remind participants to perform the novel planned healthy behaviour in the habit acquisition phase ^‡^
**Review behaviour goal(s)**	Review behaviour goal(s) jointly with the person and consider modifying goal(s) or behaviour change strategy in light of achievement. This may lead to re-setting the same goal, a small change in that goal or setting a new goal instead of (or in addition to) the first, or no change	Reviewed habit goals after each subsequent study visit ^†,‡^
**Self-monitoring of behaviour**	Establish a method for the person to monitor and record their behaviour(s) as part of a behaviour change strategy	Tracking sheets were provided for self-monitoring purposes ^‡^
**Self-monitoring of outcome(s) of behaviour**	Establish a method for the person to monitor and record the outcome(s) of their behaviour as part of a behaviour change strategy	Anthropometry and other health outcomes were assessed at each study visit ^†^

^†^ BCT primary mode of delivery—face to face. ^‡^ BCT primary mode of delivery—written.

**Table 3:** Baseline characteristics of all participants and by randomisation group.

Characteristic	All Participants (*n* = 54)	Intervention Group (*n* = 27)	Control Group (*n* = 27)
**Socio-demographic**	*n* (%)	*n* (%)	*n* (%)
**Sex (Female)**	22 (40.7)	11 (40.7)	11 (40.7)
Age (years) mean (SD) *	72.2 (5.8)	71.3 (5.6)	73.1 (6.0)
Years in full time education, mean (SD) *	13.2 (3.9)	13.3 (3.4)	13.0 (4.3)
**Occupational status**			
Working	10 (18.5)	4 (14.8)	6 (22.2)
Retired	44 (81.5)	23 (85.2)	21 (77.8)
**Multiple deprivation score (MDS)**			
1—most deprived	5 (9.3)	3 (11.1)	2 (7.4)
2	3 (5.6)	1 (3.7)	2 (7.4)
3	5 (9.3)	2 (7.4)	3 (11.1)
4	15 (27.8)	6 (22.2)	9 (33.3)
5—least deprived	26 (48.1)	15 (55.6)	11 (40.7)
**Physical and clinical measurements**			
BMI (kg/m^2^) * (*n* = 52)			
Healthy	12 (23.1)	4 (15.4)	8 (30.8)
Overweight	20 (38.5)	10 (38.5)	10 (38.5)
Obesity	20 (38.5)	12 (46.1)	8 (31.8)
Blood pressure (mmHg) *			
Systolic	146.4 (19.1)	147.6 (17.6)	145.0 (20.9)
Diastolic	82.1 (11.9)	83.8 (10.9)	80.4 (12.9)
**Health and lifestyle**			
**Smoking status** (*n* = 53)			
Current	3 (5.7)	2 (7.7)	1 (3.7)
Previous	24 (45.3)	15 (57.7)	9 (33.3)
Never	26 (49.1)	9 (34.6)	17 (63)
**Alcohol consumption**			
Never or occasionally	36 (66.7)	18 (66.7)	18 (66.7)
Once or twice a week	11 (20.4)	6 (22.2)	5 (18.5)
Three to five times a week	3 (5.6)	1 (3.7)	2 (7.4)
Six or seven times a week	4 (7.4)	2 (7.4)	2 (7.4)
**Takes nutritional supplements (yes)**	29 (53.7)	16 (59.3)	13 (48.1)
**Medication use**			
No medication	9 (16.7)	3 (11.1)	6 (22.2)
One to four medications	21 (38.9)	12 (44.4)	9 (33.3)
Five to nine medications	17 (31.5)	9 (33.3)	8 (29.6)
Ten+ medications	7 (13.0)	3 (11.1)	4 (14.8)
**Oral Rehabilitation**			
Removal partial denture	36 (66.7)	20 (74.1)	16 (59.3)
Functional dentition	18 (33.3)	7 (25.9)	11 (40.7)

Data presented as frequencies (%) or * mean (SD); (*n*) specified where it deviates from full baseline sample (*n* = 54).

**Table 4:** Mean change in generic automaticity scores for three dietary behaviours by randomisation group.

Dietary Behaviour	Group	Baseline	Change from Baseline to 6 Weeks	Difference between Groups at 6 Weeks		Change from Baseline to 4 Months	Difference between Groups at 4 Months ^¥^		Change from Baseline to 8 Months	Difference between Groups at 8 Months	
		Mean (SD)	Mean (SD)	Mean (95%CI)	*p*	Mean (SD)	Mean (95%CI)	*p*	Mean (SD)	Mean (95%CI)	*p*
**Eating ≥3 portions of fruit/veg daily**											
	IG	3.8 (3.0)	2.7 (2.6) ***			2.2 (2.8) ***			2.5 (3.0) ***		
	CG	3.5 (3.2)	0.7 (2.2)	−2.0 (−3.4, −0.6)	0.007	0.3 (1.8)	**−1.9** **(−3.3, −0.6)**	**0.005**	0.5 (3.0)	−2.0 (-3.6, −0.3)	0.020
**Choosing wholegrain sources over white alternatives**											
	IG	3.4 (2.28)	3.1 (2.0) ***			2.8 (2.2) ***			2.6 (2.0) ***		
	CG	4.0 (2.28)	0.3 (1.5)	−2.8 (−3.8, −1.8)	<0.001	0.2 (2.4)	**−2.6** **(−3.9, −1.2)**	**<0.001**	0.4 (2.3)	−2.2 (-3.4, −0.9)	0.001
**Choosing healthier protein sources over red/processed meats**											
	IG	4.4 (1.5)	1.7 (1.4) ***			1.4 (2.2) **			1.6 (1.6) ***		
	CG	4.9 (1.5)	0.3 (1.5)	−1.4 (−2.3, −0.6)	0.002	0.1 (1.8)	**−1.3** **(−2.4, −0.2)**	**0.027**	0.1 (1.8)	−1.5 (-2.5, −0.5)	0.004

Within-group (intervention and control) differences were analysed using paired-samples *t*-test (** *p* < 0.01, *** *p* < 0.001). Differences between groups (intervention vs. Control) were analysed using Independent-samples *t*-tests (*p* values shown in table). Primary outcome values are in bold. ^¥^ Note a sensitivity analysis was also conducted with baseline BMI as a covariate (ANCOVA) and the pattern of results was unchanged for differences between the groups at 4 months (primary endpoint). Sample size: IG = 22 and CG = 27 (for the change from baseline to 6 weeks analysis, 21 participants in IG were included). Abbreviations: IG, intervention group; CG, control group.

**Table 5:** Mean change in specific automaticity scores for individually tailored planned dietary behaviours within the intervention group.

Dietary Behaviour	Baseline	Change from Baseline to 6 Weeks		Change from Baseline to 4 Months		Change from Baseline to 8 Months	
	Mean (SD)	Mean (95% CI)	*p*	Mean (95% CI)	*p*	Mean (95% CI)	*p*
**Fruit and veg habit**	0.4 (1.2)	5.5 (4.8, 6.2)	<0.001	5.0 (3.8, 6.1)	<0.001	5.1 (4.0, 6.1)	<0.001
**Wholegrain habit**	1.1 (2.3)	4.7 (3.6, 5.8)	<0.001	4.1 (2.7, 5.4)	<0.001	3.7 (2.4, 5.1)	<0.001
**Healthy protein habit**	0.2 (1.0)	4.3 (3.3, 5.3)	<0.001	4.7 (3.6, 5.8)	<0.001	4.7 (3.7, 5.7)	<0.001

Changes in automaticity scores across the study time points were analysed using paired-samples *t*-tests. Sample size: IG = 22 (for the change from baseline to 4 months analysis, 21 participants were included.

**Table 6:** Mean change in MNA total scores by randomisation group.

Group	Baseline		Change from Baseline to 6 Weeks		Change from Baseline to 4 Months		Change from Baseline to 8 Months	
	Mean (SD)	*p*	Mean (SD)	*p*	Mean (SD)	*p*	Mean (SD)	*p*
**IG**	27.1 (2.4)	0.98	0.89 (1.88)	0.03	0.59 (2.50)	0.30	0.40 (2.91)	0.56
**CG**	27.1 (2.3)		−0.27 (1.69)		0.00 (1.39)		0.02 (1.60)	
**Sensitivity analysis**								
**IG**	27.3 (2.2)	0.76	0.83 (1.91)	0.04	0.95 (1.88)	0.049	0.93 (1.71)	0.07
**CG**	27.1 (2.3)		−0.27 (1.69)		0.00 (1.39)		0.02 (1.60)	

Differences between groups (intervention vs. Control) were analysed using Independent-samples *t*-tests. Sample sizes: change from baseline to 6 weeks, IG (*n* = 22) and CG (*n* = 26); change from baseline to 4 months, IG (*n* = 22) and CG (*n* = 27); change from baseline to 8 months, IG (*n* = 21) and CG (*n* = 27). One participant from the IG was excluded for sensitivity analysis. Abbreviations: IG, intervention group; CG, control group.

## Data Availability

All reasonable requests for data will be considered by contacting the Corresponding Author.

## Supplementary Materials

The following supporting information can be downloaded at: https://www.mdpi.com/article/10.3390/nu15030731/s1, Table S1: Mean daily nutrient intake differences between randomisation groups; Table S2: Mean Daily Micronutrient Intake Differences Between Randomization Groups.

## References

[1] Fávaro-Moreira N.C., Krausch-Hofmann S., Matthys C., Vereecken C., Vanhauwaert E., Declercq A., Bekkering G.E., Duyck J. (2016). Risk Factors for Malnutrition in Older Adults: A Systematic Review of the Literature Based on Longitudinal Data. Adv. Nutr..

[2] Toniazzo M.P., Amorim P.d.S., Muniz F.W.M.G., Weidlich P. (2018). Relationship of nutritional status and oral health in elderly: Systematic review with meta-analysis. Clin. Nutr..

[3] Tada A., Miura H. (2014). Systematic review of the association of mastication with food and nutrient intake in the independent elderly. Arch. Gerontol. Geriatr..

[4] Watson S., McGowan L., McCrum L.A., Cardwell C.R., McGuinness B., Moore C., Woodside J.V., McKenna G. (2019). The impact of dental status on perceived ability to eat certain foods and nutrient intakes in older adults: Cross-sectional analysis of the UK National Diet and Nutrition Survey 2008–2014. Int. J. Behav. Nutr. Phys. Act..

[5] McKenna G., Allen P.F., O’Mahony D., Flynn A., Cronin M., DaMata C., Woods N. (2014). Comparison of functionally orientated tooth replacement and removable partial dentures on the nutritional status of partially dentate older patients: A randomised controlled clinical trial. J. Dent..

[6] McGowan L., Mccrum L.-A., Watson S., Cardwell C., McGuinness B., Rutherford H., Paice V., Moore C., Brocklehurst P.R., Woodside J.V. (2020). The impact of oral rehabilitation coupled with healthy dietary advice on the nutritional status of adults: A systematic review and meta-analysis. Crit. Rev. Food Sci. Nutr..

[7] Reinders I., Volkert D., de Groot L.C.P.G.M., Beck A.M., Feldblum I., Jobse I., Neelemaat F., de van der Schueren M.A., Shahar D.R., Smeets E.T. (2018). Effectiveness of nutritional interventions in older adults at risk of malnutrition across different health care settings: Pooled analyses of individual participant data from nine randomized controlled trials. Clin. Nutr..

[8] Batsis J.A., Gill L.E., Masutani R.K., Adachi-Mejia A.M., Blunt H.B., Bagley P.J., Lopez-Jimenez F., Bartels S.J. (2017). Weight Loss Interventions in Older Adults with Obesity: A Systematic Review of Randomized Controlled Trials Since 2005. J. Am. Geriatr. Soc..

[9] Fjeldsoe B., Neuhaus M., Winkler E., Eakin E. (2011). Systematic review of maintenance of behavior change following physical activity and dietary interventions. Health Psychol..

[10] Rothman A.J. (2000). Toward a theory-based analysis of behavioral maintenance. Health Psychol..

[11] Lally P., van Jaarsveld C.H.M., Potts H.W.W., Wardle J. (2010). How are habits formed: Modelling habit formation in the real world. Eur. J. Soc. Psychol..

[12] Gardner B. (2015). A review and analysis of the use of “habit” in understanding, predicting and influencing health-related behaviour. Health Psychol. Rev..

[13] Kwasnicka D., Dombrowski S.U., White M., Sniehotta F. (2016). Theoretical explanations for maintenance of behaviour change: A systematic review of behaviour theories. Health Psychol. Rev..

[14] McGowan L., Cooke L.J., Gardner B., Beeken R.J., Croker H., Wardle J. (2013). Healthy feeding habits: Efficacy results from a cluster-randomized, controlled exploratory trial of a novel, habit-based intervention with parents. Am. J. Clin. Nutr..

[15] A Carels R., Burmeister J.M., Koball A.M., Oehlhof M.W., Hinman N., LeRoy M., Bannon E., Ashrafioun L., Storfer-Isser A., A Darby L. (2014). A randomized trial comparing two approaches to weight loss: Differences in weight loss maintenance. J. Health Psychol..

[16] Beeken R.J., Leurent B., Vickerstaff V., Wilson R., Croker H., Morris S., Omar R.Z., Nazareth I., Wardle J. (2017). A brief intervention for weight control based on habit-formation theory delivered through primary care: Results from a randomised controlled trial. Int. J. Obes..

[17] Mergelsberg E.L.P., Mullan B.A., Allom V., Scott A. (2021). An intervention designed to investigate habit formation in a novel health behaviour. Psychol. Health.

[18] Wood W., Neal D.T. (2009). The habitual consumer. J. Consum. Psychol..

[19] Wansink B., Sobal J. (2007). Mindless Eating. Environ. Behav..

[20] Lally P., Gardner B. (2013). Promoting habit formation. Health Psychol. Rev..

[21] Gardner B.D., Rebar A.L. (2019). Habit Formation and Behavior Change.

[22] Zhou X., Perez-Cueto F.J.A., Dos Santos Q., Monteleone E., Giboreau A., Appleton K.M., Bjørner T., Bredie W.L.P., Hartwell H. (2018). A Systematic Review of Behavioural Interventions Promoting Healthy Eating among Older People. Nutrients.

[23] Hale J.M., Schneider D.C., Gampe J., Mehta N.K., Myrskylä M. (2020). Trends in the risk of cognitive impairment in the United States, 1996–2014. Epidemiology.

[24] Matei R., Thuné-Boyle I., Hamer M., Iliffe S., Fox K.R., Jefferis B.J., Gardner B. (2015). Acceptability of a theory-based sedentary behaviour reduction intervention for older adults (‘On Your Feet to Earn Your Seat’). BMC Public Health.

[25] Eldridge S.M., Chan C.L., Campbell M.J., Bond C.M., Hopewell S., Thabane L., Lancaster G.A. (2016). CONSORT 2010 statement: Extension to randomised pilot and feasibility trials. Pilot Feasib. Stud..

[26] Hoffmann T.C., Glasziou P.P., Boutron I., Milne R., Perera R., Moher D., Altman D.G., Barbour V., Macdonald H., Johnston M. (2014). Better reporting of interventions: Template for intervention description and replication (TIDieR) checklist and guide. BMJ.

[27] Craig P., Dieppe P., Macintyre S., Michie S., Nazareth I., Petticrew M. (2013). Developing and evaluating complex interventions: The new Medical Research Council guidance. Int. J. Nurs. Stud..

[28] McCrum L.A., Watson S., McGowan L., McGuinness B., Cardwell C., Clarke M., Woodside J.V., McKenna G. (2020). Development and feasibility of a tailored habit-based dietary intervention coupled with natural tooth replacement on the nutritional status of older patients. Pilot Feasib. Stud..

[29] Public Health England (2016). The Eatwell Guide. https://www.gov.uk/government/publications/the-eatwell-guide.

[30] Michie S., Richardson M., Johnston M., Abraham C., Francis J., Hardeman W., Eccles M.P., Cane J., Wood C.E. (2013). The Behavior Change Technique Taxonomy (v1) of 93 Hierarchically Clustered Techniques: Building an International Consensus for the Reporting of Behavior Change Interventions. Ann. Behav. Med..

[31] Gardner B., Abraham C., Lally P., de Bruijn G.-J. (2012). Towards parsimony in habit measurement: Testing the convergent and predictive validity of an automaticity subscale of the Self-Report Habit Index. Int. J. Behav. Nutr. Phys. Act..

[32] Vellas B., Guigoz Y., Garry P.J., Nourhashemi F., Bennahum D., Lauque S., Albarede J.-L. (1999). The mini nutritional assessment (MNA) and its use in grading the nutritional state of elderly patients. Nutrition.

[33] (2018). NISRA NIMDM17-SA Level Results|Northern Ireland Statistics and Research Agency. https://www.nisra.gov.uk/publications/nimdm17-sa-level-results.

[34] Cleo G., Glasziou P., Beller E., Isenring E., Thomas R. (2019). Habit-based interventions for weight loss maintenance in adults with overweight and obesity: A randomized controlled trial. Int. J. Obes..

[35] Verplanken B. (2006). Beyond frequency: Habit as mental construct. Br. J. Soc. Psychol..

[36] Lally P., Chipperfield A., Wardle J. (2008). Healthy habits: Efficacy of simple advice on weight control based on a habit-formation model. Int. J. Obes..

[37] Gardner B., Phillips L.A., Judah G. (2016). Habitual instigation and habitual execution: Definition, measurement, and effects on behaviour frequency. Br. J. Health Psychol..

[38] Lara J., Evans E.H., O’Brien N., Moynihan P.J., Meyer T.D., Adamson A.J., Errington L., Sniehotta F.F., White M., Mathers J.C. (2014). Association of behaviour change techniques with effectiveness of dietary interventions among adults of retirement age: A systematic review and meta-analysis of randomised controlled trials. BMC Med..

[39] Peng W., Li L., Kononova A., Cotten S., Kamp K., Bowen M. (2021). Habit Formation in Wearable Activity Tracker Use Among Older Adults: Qualitative Study. JMIR mHealth uHealth.

[40] Rossi A.P., Rubele S., Zamboni M., Walrand S. (2019). Chapter 6—Sarcopenic Obesity. Nutrition and Skeletal Muscle.

[41] Volpp K.G., Loewenstein G. (2020). What is a habit? Diverse mechanisms that can produce sustained behavior change. Organ. Behav. Hum. Decis. Process..

[42] Hamirudin A.H., Charlton K., Walton K. (2016). Outcomes related to nutrition screening in community living older adults: A systematic literature review. Arch. Gerontol. Geriatr..

[43] Morais J.A., Chevalier S., Gougeon R. (2006). Protein turnover and requirements in the healthy and frail elderly. J. Nutr. Health Aging.

[44] Bradbury J., Thomason J.M., Jepson N.J.A., Walls A.W.G., Allan P.F., Moynihan P.J. (2006). Nutrition counseling increases fruit and vegetable consumption in the edentulous. J. Dent. Res..

[45] Moynihan P., Thomason M., Walls A., Gray-Donald K., Morais J.A., Ghanem H., Wollin S., Ellis J., Steele J., Lund J. (2009). Researching the impact of oral health on diet and nutritional status: Methodological issues. J. Dent..

[46] Kotronia E., Brown H., Papacosta A., Lennon L., Weyant R., Whincup P., Ramsay S. (2021). Poor oral health and the association with diet quality and intake in older people in two studies in the UK and USA. Br. J. Nutr..

[47] Hagger M.S., Rebar A.L., Mullan B., Lipp O.V., Chatzisarantis N.L. (2015). The subjective experience of habit captured by self-report indexes may lead to inaccuracies in the measurement of habitual action. Health Psychol. Rev..

[48] Labrecque J.S., Wood W. (2015). What measures of habit strength to use? Comment on Gardner. Health Psychol. Rev..

[49] Census 2021: Main Statistics for Northern Ireland Statistical Bulletin Ethnic Group 22 September 2022. https://www.nisra.gov.uk/system/files/statistics/census-2021-main-statistics-for-northern-ireland-phase-1-statistical-bulletin-ethnic-group.pdf.

[50] Buys D.R., Francis S.L., Marra M.V., Locher J.L., Lofgren I.E. (2020). Lessons Learned: Recruiting Aging Adults for Research. Top. Clin. Nutr..

[51] Giskes K., Avendaňo M., Brug J., Kunst A.E. (2010). A systematic review of studies on socioeconomic inequalities in dietary intakes associated with weight gain and overweight/obesity conducted among European adults. Obes. Rev..

