# Supplementary material for: Establishing Healthy Eating ‘Habits’: A Pilot Randomised Controlled Trial of a Habit-Based Dietary Intervention following Oral Rehabilitation for Older Adults
Source: Nutrients. 2023 Feb 1;15(3):731. doi: 10.3390/nu15030731 (PMC9919723; doi:10.3390/nu15030731)
Supplement: Supplementary file 1 [file nutrients-15-00731-s001.zip › nutrients-2141813-supplementary.pdf]

Supplementary Table S1: Mean daily nutrient intake differences between randomisation groups

Nutrient	Group	Baseline	Change from baseline to 6 weeks	Difference between groups at 6 weeks		Change from baseline to 4 months	Difference between groups at 4 months		Change from baseline to 8 months	Difference between groups at 8 months	
		Mean (SD)	Mean (SD)	Mean (95%CI)	*P*	Mean (SD)	Mean (95%CI)	*P*	Mean (SD)	Mean (95%CI)	*P*
**Energy kcal/d**	IG	1613.32 (416.94)	57.58 (454.52)			39.85 (599.44)			31.87 (631.35)		
	CG	1661.18 (460.92)	38.91 (267.53)	-18.67 (-254.55, 217.20)	0.87	-12.21 (207.48)	-52.06 (-393.70, 289.58)	0.75	-114.36 (304.88)	-146.23 (-515.16, 222.70)	0.42
**Protein g/d**	IG	67.48 (19.24)	11.65 (21.43)			4.78 (21.13)			3.32 (26.51)		
	CG	73.25 (16.10)	-0.15 (12.70)	-11.80 (-23.88, 0.28)	0.06	-1.77 (15.01)	-6.55 (-18.96, 5.86)	0.29	-6.10 (11.80)	-9.41 (-24.78, 5.95)	0.21
**Fat g/d**	IG	63.65 (20.03)	-2.35 (27.33)			-0.87 (31.39)			-0.51 (30.42)		
	CG	65.01 (18.98)	5.38 (17.71)	7.73 (-6.91, 22.38)	0.29	-0.45 (12.22)	0.42 (-17.61, 18.45)	0.96	-5.32 (16.87)	-4.81 (-22.89, 13.27)	0.59
**Sat Fat g/d**	IG	24.78 (9.09)	-0.36 (9.96)			0.29 (10.97)			0.33 (10.85)		
	CG	24.97 (8.20)	2.17 (8.91)	2.53 (-3.61, 8.67)	0.41	-0.49 (4.84)	-0.77 (-7.14, 5.60)	0.80	-1.23 (7.00)	-1.56 (-8.17, 5.06)	0.63
**Mono Fat g/d**	IG	22.74 (7.67)	-1.84 (9.86)			-0.45 (12.76)			-0.43 (12.59)		
	CG	22.56 (7.16)	1.22 (7.54)	3.06 (-2.58, 8.70)	0.28	-0.78 (5.77)	-0.33 (-7.75, 7.10)	0.93	-2.21 (7.78)	-1.77 (-9.39, 5.84)	0.63
**Omega 3 FA g/d**	IG	1.37 (1.18)	-0.34 (1.37)			-0.42 (1.47)			-0.26 (1.91)		
	CG	1.33 (0.85)	0.29 (1.52)	0.62 (-0.33, 1.58)	0.19	0.02 (1.18)	0.45 (-0.47, 1.36)	0.33	1.30 (0.28)	0.24 (-0.84, 1.33)	0.65
**Omega 6 FA g/d**	IG	4.60 (2.49)	-1.11 (2.79)			-0.43 (3.24)			-0.57 (3.16)		
	CG	4.92 (2.50)	0.09 (3.42)	1.20 (-0.87, 3.27)	0.25	-0.42 (2.97)	0.01 (-2.14, 2.15)	0.99	-1.08 (2.55)	-0.51 (-2.45, 1.43)	0.60
**Carbohydrate g/d**	IG	177.16 (47.53)	9.78 (54.63)			4.24 (73.15)			7.09 (83.40)		
	CG	181.19 (74.64)	2.51 (39.08)	-7.28 (-39.34, 24.79)	0.65	3.12 (40.70)	-1.12 (-44.80, 42.56)	0.96	-5.90 (48.09)	-12.99 (-57.64, 31.67)	0.56

Nutrient	Group	Baseline	Change from baseline to 6 weeks	Difference between groups at 6 weeks		Change from baseline to 4 months	Difference between groups at 4 months		Change from baseline to 8 months	Difference between groups at 8 months	
		**Mean (SD)**	**Mean (SD)**	**Mean (95%CI)**	***P***	**Mean (SD)**	**Mean (95%CI)**	***P***	**Mean (SD)**	**Mean (95%CI)**	***P***
**Free sugars g/d**	IG	35.57 (17.15)	-8.15 (20.27)			-4.93 (25.46)			-10.15 (21.51)		
	CG	35.04 (23.70)	-1.84 (20.27)	6.31 (-6.38, 19.01)	0.32	-4.43 (15.40)	0.50 (-14.91, 15.91)	0.95	-9.86 (14.29)	0.30 (-11.81, 12.40)	0.96
**Fibre g/d**	IG	17.00 (6.04)	4.06 (7.11)			3.46 (8.07)	-2.10 (-6.86, 2.66)	0.37	1.87 (9.07)		
	CG	17.80 (7.94)	0.27 (4.27)	-3.78 (-7.80, 0.23)	0.06	1.35 (4.12)			0.51 (6.81)	-1.36 (-6.73, 4.01)	0.61

Differences between groups (intervention vs. Control) are analysed using Independent sample t-tests. Sample sizes: Change from baseline to 6 weeks, IG (n=17) and CG (n=22); change from baseline to 4 months, IG (n=15) and CG (n=20); change from baseline to 8 motnhs, IG (n=15) and CG (n=21). Abbreviations: IG, intervention group; CG, control group; sat, saturated, mono, monounsaturated; FA, fatty acid.

Supplementary Table S2: Mean Daily Micronutrient Intake Differences Between Randomization Groups

Nutrient	Group	Baseline	Change from baseline to 6 weeks	Difference between groups at 6 weeks	*P*	Change from baseline to 4 months	Difference between groups at 4 months	*P*	Change from baseline to 8 months	Difference between groups at 8months	*P*
		Mean (SD)	Mean (SD)	Mean (95%CI)		Mean (SD)	Mean (95%CI)		Mean (SD)	Mean (95%CI)	
**Vitamin A μg/d**	IG	926.54 (480.13)	56.42 (617.14)			-6.73 (692.96)			68.40 (1223.40)		
	CG	967.20 (791.47)	529.78 (1579.24)	473.35 (-349.14, 1295.85)	0.25	211.58 (1264.66)	218.30 (-518.63, 955.24)	0.55	-231.07 (757.49)	-299.47 (-970.44, 371.50)	0.37
**Vitamin B6 μg/d**	IG	2.30 (2.35)	0.55 (1.68)			7.02 (24.98)			2.52 (10.44)		
	CG	2.28 (1.64)	0.30 (2.15)	-0.25 (-1.53, 1.03)	0.70	0.05 (0.73)	-6.97 (-20.81, 6.86)	0.30	-0.54 (1.31)	-3.07 (-8.86, 2.74)	0.28
**Vitamin B12 μg/d***	IG	6.08 (6.12)	17.55 (75.23)			0.54 (4.93)			-0.02 (8.90)		
	CG	4.75 (2.16)	1.87 (6.45)	-15.68 (-54.43, 23.08)	0.40	0.78 (4.35)	0.25 (-2.95, 3.45)	0.88	0.12 (2.60)	0.14 (-4.88, 5.17)	0.95
**Folate μg/d**	IG	246.27 (129.39)	90.95 (164.21)			167.85 (331.03)			-14.73 (146.35)		
	CG	321.22 (284.97)	12.07 (108.49)	-31.001 (-227.85, 165.84)	0.75	33.34 (103.67)	-134.50 (-322.18, 53.17)	0.15	-50.43 (170.77)	-35.70 (-146.24, 75.02)	0.52
**Vitamin C mg/d**	IG	121.62 (222.73)	12.07 (108.49)			23.27 (193.40)			22.34 (239.53)		
	CG	109.91 (80.75)	54.81 (94.79)	-42.73 (-109.99, 24.52)	0.21	-4.41 (67.05)	-27.68 (-122.08, 66.73)	0.56	-13.42 (70.38)	-35.77 (-147.69, 76.15)	0.52
**Vitamin D μg/d***	IG	4.36 (3.24)	-0.36 (3.69)			-1.43 (3.29)			-0.87 (3.60)		
	CG	5.34 (4.75)	2.75 (11.49)	3.11 (-2.77, 8.99)	0.29	1.08 (5.86)	2.52 (-0.92, 5.95)	0.15	0.47 (11.01)	1.34 (-4.67, 7.35)	0.65
**Iron mg/d**	IG	10.18 (4.06)	0.49 (3.06)			0.45 (4.06)			-0.14 (5.25)		
	CG	11.23 (4.97)	3.49 (22.13)	3.00 (-7.98, 13.99)	0.58	1.79 (11.69)	1.34 (-5.09, 7.77)	0.68	-0.77 (2.77)	-0.63 (-3.37, 2.10)	0.64
**Calcium mg/d**	IG	746.61 (298.92)	228.60 (372.80)			156.43 (369.29)			95.10 (366.05)		
	CG	834.96 (401.72)	89.74 (224.43)	-138.86 (-349.66, 71.94)	0.19	-15.83 (164.88)	-172.26 (-386.86, 42.35)	0.11	-146.48 (340.82)	-241.58 (-483.02, -0.14)	0.05

**Magnesium mg/d**	IG	261.94 (85.97)	59.07 (87.40)			50.75 (118.48)			25.34 (118.90)		
	CG	271.80 (89.87)	33.08 (130.09)	-25.99 (-100.32, 48.35)	0.48	30.14 (85.22)	-20.61 (-90.58, 49.35)	0.55	3.42 (74.31)	-21.93 (-87.35, 43.50)	0.50
**Potassium mg/d**	IG	2742.37 (761.63)	517.11 (776.61)			502.78 (1078.21)			221.70 (1155.55)		
	CG	2763.02 (798.57)	64.71 (453.89)	-452.40 (-889.19, -15.61)	**0.04**	-41.48 (523.92)	-544.25 (-1176.52, 88.02)	0.09	-40.77 (774.69)	-262.47 (321.22, 390.33)	0.42
**Iodine μg/d**	IG	138.53 (81.60)	44.38 (113.69)			30.34 (86.26)			10.40 (100.52)		
	CG	155.38 (82.56)	36.26 (245.92)	-8.12 (-138.84, 122.59)	0.89	8.98 (135.05)	-21.36 (-102.57, 59.85)	0.60	-11.85 (79.63)	-22.25 (-83.27, 38.78)	0.46

Differences between groups (intervention vs. Control) are analysed using Independent sample t-tests. Change from baseline to 6 weeks, IG (n=17) and CG (n=22); change from baseline to 4 months, IG (n=15) and CG (n=20); change from baseline to 8 motnhs, IG (n=15) and CG (n=21).Abbreviations: IG, intervention group; CG, control group.
